# Patients’ Experiences of the Western HealthLinks Integrated Care Chronic Illness Management Program: A Prospective Mixed-Methods Observational Cohort Study

**DOI:** 10.5334/ijic.8980

**Published:** 2026-09-07

**Authors:** Julia L. Jones, Kirsty Barnes, Catherine Grant, Jason Plant, Natalie G. Lumsden, Koen Simons, Edward D. Janus, Craig L. Nelson, Ann Allenby

**Affiliations:** 1 Western Health, Western Health Chronic Disease Alliance, University of Melbourne, Australia; 2 Western Health, Western Health Chronic Disease Alliance, Australia; 3 Western Health, University of Melbourne, Australia; 4 Western Health, Australia

**Keywords:** chronic disease, complex care needs, integrated care, coordinated care, Quality of Life, AQoL

## Abstract

**Introduction:**

Western HealthLinks, an integrated care chronic illness management program, supported patients with chronic and complex conditions to stay well at home after hospital discharge. This study assessed patients’ experiences of the program.

**Methods:**

A prospective mixed-methods observational cohort study, conducted as an implementation evaluation with six-monthly assessments over 30 months included: A visual analogue scale (VAS) rating healthcare satisfaction; an Assessment of Quality of Life 4 Dimensions (AQoL4D) tool; a survey regarding healthcare satisfaction. VAS and AQoL4D were analysed with a simple random effects model using time as a linear variable; survey was analysed with thematic analysis.

**Results:**

Median baseline VAS was 79 and AQoL4D 0.15, with no evidence for change over time. Themes across timepoints included: Local/home-based care; hospital access; individualised, coordinated and consistent care; communication; reduced waiting; healthcare provider qualities; transport/parking.

**Discussion:**

No evidence of change to VAS and AQoL4D suggests maintained healthcare satisfaction and QoL over time despite increasing age and disease burden. Surveys showed patients value integrated care. Limitations include the absence of control group and loss to follow-up with risk of bias for VAS and AQoL4D.

**Conclusion:**

Western HealthLinks patients maintained healthcare satisfaction and QoL over time after hospital discharge. Patients value integrated care and future models of care may benefit from incorporation of themes from analysis.

## Introduction

Fragmented care can occur when patients are moving from hospital to home, which can lead to adverse outcomes [1] as well as impacting health care experience [2] and quality of life (QoL) [3].

Integrated care has been defined in a number of ways, including by National Voices, the coalition of health and social care charities in England, provided in “A Narrative for Person-Centred Coordinated Care” which considers that “integrated care” is equivalent to “person centred coordinated care” which is summed up by the statement “I can plan my care with people who work together to understand me and my carer(s), allow me control, and bring together services to achieve the outcomes important to me” [4].

Systematic review evidence has found that integrated care based interventions can increase patient satisfaction and perceived quality of care [5]. However much of the data regarding integrated care relate to programs targeting individual diseases rather than patients with multi-morbidity or those with chronic and complex care needs [6]. Additionally, many of the studies assessing integrated care come from Europe and North America [5] and their findings cannot always be generalised to different populations and healthcare systems.

There is some Australian data regarding integrated care-based interventions, however there is marked heterogeneity in intervention setting, components and outcomes. Most studies did not assess patient experience, but one qualitative study that did so found an improvement in patient and carer experience with the integrated care based intervention [7]. Of those Australian studies that assessed QoL one randomised controlled trial found no improvement with the intervention [8] while another quasi-experimental study found improved QoL for the subset of patients with heart failure [9].

This variability in health outcomes is highlighted in an umbrella review of systematic reviews assessing the impact of integrated care on QoL for patients with chronic conditions, which found only half of the reviews assessing discharge management interventions demonstrated benefits for patient QoL [10]. It is therefore important to evaluate the patients’ experiences and assess health related quality of life (HRQoL) when providing new models of care.

The aim of this study was to assess the patients’ experiences of the Western HealthLinks intervention and HRQoL. The expectation was that without intervention, satisfaction with healthcare and HRQoL would decline over time with increasing patient age and disease burden. Western HealthLinks sought to modify this trajectory to help patients maintain healthcare satisfaction and HRQoL. This study used a prospective mixed-methods observational cohort study, conducted as an implementation evaluation to assess patients’ experiences of an integrated care-based model, Western HealthLinks, and assess their QoL over time.

## Methods

### Setting and Context

Starting in 2016, the State Government of Victoria enabled health services to use a flexible funding model in which projected inpatient funding could be used to provide care to patients at high risk of multiple hospital readmissions. Hospitals could develop models to support these patients to manage their health conditions at home, thereby reducing hospitalisations without additional government spending.

A digital algorithm [11] was developed by the Victorian Department of Health and Human Services (DHHS) to identify patients at high risk of hospital readmission. The algorithm was designed to exclude episodes of care that would not be funded by DHHS, episodes that are likely not preventable and of high cost and patients whose care is covered by specialist state-wide programs. A Flowchart outlining the process for determining Western HealthLinks eligibility and eligibility for this patient experience study is presented in Supplementary Materials Figure 1. Once a patient had an unplanned “trigger admission”, which identified them as being HealthLinks eligible, their next unplanned admission would be funded by a capitation grant based on projected inpatient activity-based funding. If the patient did not consent to the Western HealthLinks program, the hospital would still receive the capitation grant in place of usual funding, but the patient would receive usual care. The capitation grant would cover inpatient as well as subsequent care upon return home.

Western Health is a publicly funded health service, which includes two tertiary hospitals as well as sub-acute, ambulatory, psychiatric, drug and alcohol and community-based care facilities. Western Health is in the western suburbs of Melbourne and serves a culturally and linguistically diverse community and includes many people in challenging socio-economic circumstances. At Western Health, an Integrated Care Committee was formed in 2015 which undertook a community engagement process with involvement of patients and carers, conducted case reviews and reviewed the literature on integrated care models. The committee then identified the following ten key success factors to quality care integration: 1) identification system; 2) advanced discharge program; 3) post discharge support program; 4) expert ‘hospital like’ clinical care in the home; 5) care coordination/care navigation; 6) integration with GPs and primary care; 7) post discharge pharmacy review; 8) e-health; 9) advanced care planning; 10) multicomponent integration and collaboration strategies. All these factors were used to develop the Western HealthLinks model of care.

Western HealthLinks commenced in November 2016 with the patient identification algorithm initially generated by DHHS and provided to Western Health monthly. From 1 May 2017, local health service data was used, enabling real-time identification of patients with the Western Health Performance Unit identifying potential patients. Eligible patients who consented to the program were assessed with nine questions (The Western 9) which provided a debility, psychosocial risk score designed to further assess risk of hospital readmission (see Supplementary Materials Table 1). Caregivers were involved in the assessment of risk of hospital readmission via completion of this questionnaire. This assessment stratified patients into high, medium or low risk groups which determined the level of support provided as part of the intervention (see Supplementary Materials Table 2). In addition to the supports provided to all Western HealthLinks patients, those at high risk received weekly home visits and those at medium risk received fortnightly home visits as well as fortnightly phone support. Level of risk was re-assessed at subsequent contact points with intervention staff and if readmitted to Western Health, patients were automatically reclassified as high-risk. The intervention was delivered to all consenting patients until the end of the study in November 2019.

While in hospital, a care plan was developed in consultation with the patient’s inpatient medical team and their GP. The intervention was delivered in collaboration with SilverChain, a community not-for-profit provider of in home and community-based care. Patients received support from SilverChain including care navigators and registered nurse phone support available 24 hours every day. SilverChain also provided a Priority Response and Assessment (PRA) service from 0700 until 2200 which triggered a home visit from a nurse remotely supported by the patient’s GP or a SilverChain GP. Allied health, home-based support and behavioural health support was available through Western Health’s Health Independence Program and community partners. Different components of the Western HealthLinks Model are shown in Supplementary Materials Figure 2.

### Study Methods

This was a prospective mixed-methods observational cohort study conducted as an implementation evaluation, with longitudinal data from baseline to up to 30 months. All patients who agreed to participate in the Western HealthLinks intervention were approached at the time of their enrolment and invited to be involved in the patient experience component of the evaluation. Written informed consent was obtained at the point of initial contact (see Supplementary Materials Form 1. The mixed methods approach included visual analogue scale (VAS), Assessment of Quality of Life 4 Dimensions (AQoL4D) and a patient survey (see Supplementary Materials Form 2). Participants were requested to complete the assessments at each timepoint (at baseline and after 6, 12, 18, 24 and 30 months on the program). Patients were asked to complete the survey when a HealthLinks staff member was present either by phone or face-to-face.

People with lived experience contributed to the development of the Western HealthLinks program through the community engagement process in which patients and carers provided input regarding what was important for their healthcare. This was used to inform the design of Western HealthLinks to maximise the likelihood of it meeting the requirements of patients and carers who would then use the program.

#### Measures

##### VAS

The VAS was used to assess satisfaction with healthcare. The VAS was a 100 mm line anchored at either end with 0 = “not satisfied” and 100 = “highly satisfied”. Participants marked the point on the line that best reflected their overall satisfaction. Trend over time for VAS was assessed with a Bayesian random effects model using default weakly-informative priors with time as a linear variable using R version 3.5.1 and the rstanarm library.

##### AQoL4D

Patient HRQoL was assessed with the AQoL4D instrument [12]. AQoL4D is a multi-attribute utility instrument with 12 questions which takes 1–2 minutes for patients to complete and assesses four dimensions: Independent living, mental health, relationships and senses [13]. AQoL4D scores range from –0.04 representing a state worse than death to 1 consistent with optimal HRQoL with a score of 0 representing death equivalence [14]. Trend over time for AQoL4D was assessed using a Bayesian random effects model using default weakly-informative priors with time as a linear variable using R version 3.5.1 and the rstanarm library.

##### Patient survey

The patient survey was used to assess the patients’ healthcare experiences at baseline and throughout the program. Author AA (Registered Nurse, Masters Educational Studies, Graduate Diploma Health Economics and Policy, Doctor of Nursing) developed the patient survey. The survey consisted of three questions as follows: 1) “What works well with the healthcare you receive?”; 2) “What doesn’t work well with the healthcare you receive?”; 3) “What could be done to improve the healthcare you receive?”

#### Patient Survey Qualitative Data analysis

Survey results from baseline, 6, 12, 18 and 24 months were assessed with an inductive qualitative thematic analysis using the framework outlined by Braun and Clarke [15]. Authors AA, CN, NL and JJ undertook the thematic analysis with author AA leading the process. In the first instance the researchers independently examined the patients’ responses to the survey questions which had been transcribed into an excel spreadsheet by study staff, familiarised themselves with the data and then employed open coding to create preliminary codes. Following this, at validation meetings, the independent results were discussed by the research team, the preliminary codes were written on a whiteboard and were grouped together into related concepts and mapped. Using an inductive approach, through a process of negotiation, the main themes were generated and then refined. Themes were developed by analysing codes across all questions rather than just within questions. Researchers then verified the themes to ensure they were consistent with the data and appropriately answered the research questions. After the themes were established, researches selected examples from the data to be included in the research output as quotations.

All surveys received at the time of each of the validation meetings were analysed. Due to the richness of qualitative data, once saturation (where no new themes are being generated) is achieved, there is no additional benefit in analysing further surveys. Therefore, if saturation was reached with the number of surveys assessed at each of the validation meetings, surveys received after this would not be included in the analysis. The baseline data would incorporate a large number of responses and would likely achieve saturation. Saturation in this initial analysis could be confirmed if after analysing the first half of the responses, no new themes were generated in the second half. Then, if the main themes from this initial analysis emerged at subsequent time points it was thought likely that these had also achieved saturation. Reflexivity was considered using several orientations: personal, interpersonal, methodological and contextual.

In order to achieve rigour and trustworthiness in this qualitative analysis, we have used a well-established framework for thematic analysis, methodically coded data and then after generating themes, verified them to ensure consistency with the data and coherence with the study objectives. We have also practiced reflexivity and provided transparency regarding process.

#### Ethics

This research was approved by the Western Health Research and Ethics Committee LNR/16/WH/165.

## Results

**Figure 1:**
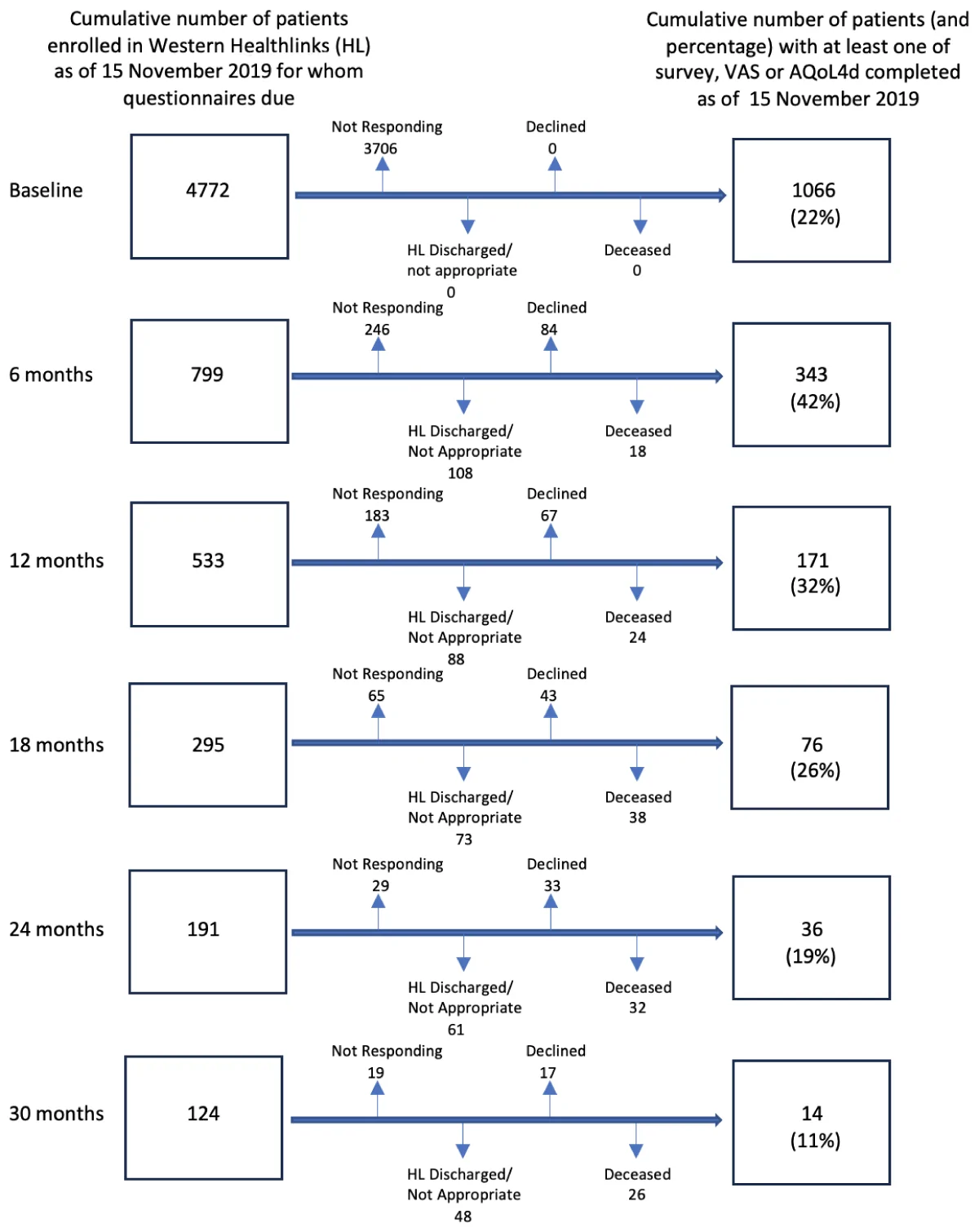
Flow chart illustrating patient enrolment in the Western HealthLinks program over time and the number who completed at least one component of the study questionnaire at each time point.

The first patient was recruited on the 21^st^ of November 2016 and the final data was collected in November 2019, providing 3 years of data for the patients recruited at the start of the study, but those recruited in June 2019 would only be able to provide data for baseline and 6-month time points. The total number of patients enrolled in the Western HealthLinks program for whom VAS/AQoL4D/survey data was due and the number who completed at least one of these tools at each time point as at November 2019 is presented in a flow chart (see Figure 1). There were missing data at some timepoints for some of these patients so the total number of included data points for each variable at different time points is lower than this. The analysis of this data is an “as treated” analysis since only those patients who received the Western HealthLinks intervention were asked to complete the study tools.

Of the 1066 patients who had completed at least one of the instruments used to collect data about their healthcare experience/HRQoL, mean age was 74.9 and median 77 years. Other demographic data including gender and country of birth for all patients who completed at least one survey are presented in Table 1.

**Table 1:** Demographic characteristics of patients who completed at least one patient survey.


CHARACTERISTIC	NUMBER (AND PERCENTAGE)

Gender*	

Female	487 (46.7%)

Male	555 (53.3%)

Age	

<46 years	28 (2.7%)

46–65 years	160 (15.4%)

66–75 years	295 (28.3%)

76–85 years	374 (35.9%)

86–95 years	169 (16.2%)

>95 years	16 (1.5%)

Country of Birth (with >1.5% of population)	

Australia	490 (47%)

Malta	93 (8.9%)

United Kingdom	74 (7.1%)

Italy	69 (6.6%)

Germany	27 (2.6%)

Croatia	26 (2.5%)

Greece	26 (2.5%)

Ireland	16 (1.5%)

Yugoslavia/Macedonia	15 (1.4%)

Philippines	15 (1.4%)

Poland	15 (1.4%)

Scotland	15 (1.4%)

New Zealand	12 (1.2%)

Others (<1.1% per country)	149 (14.3%)


*Gender as per medical record data.

### VAS (Quantitative Analysis)

**Figure 2:**
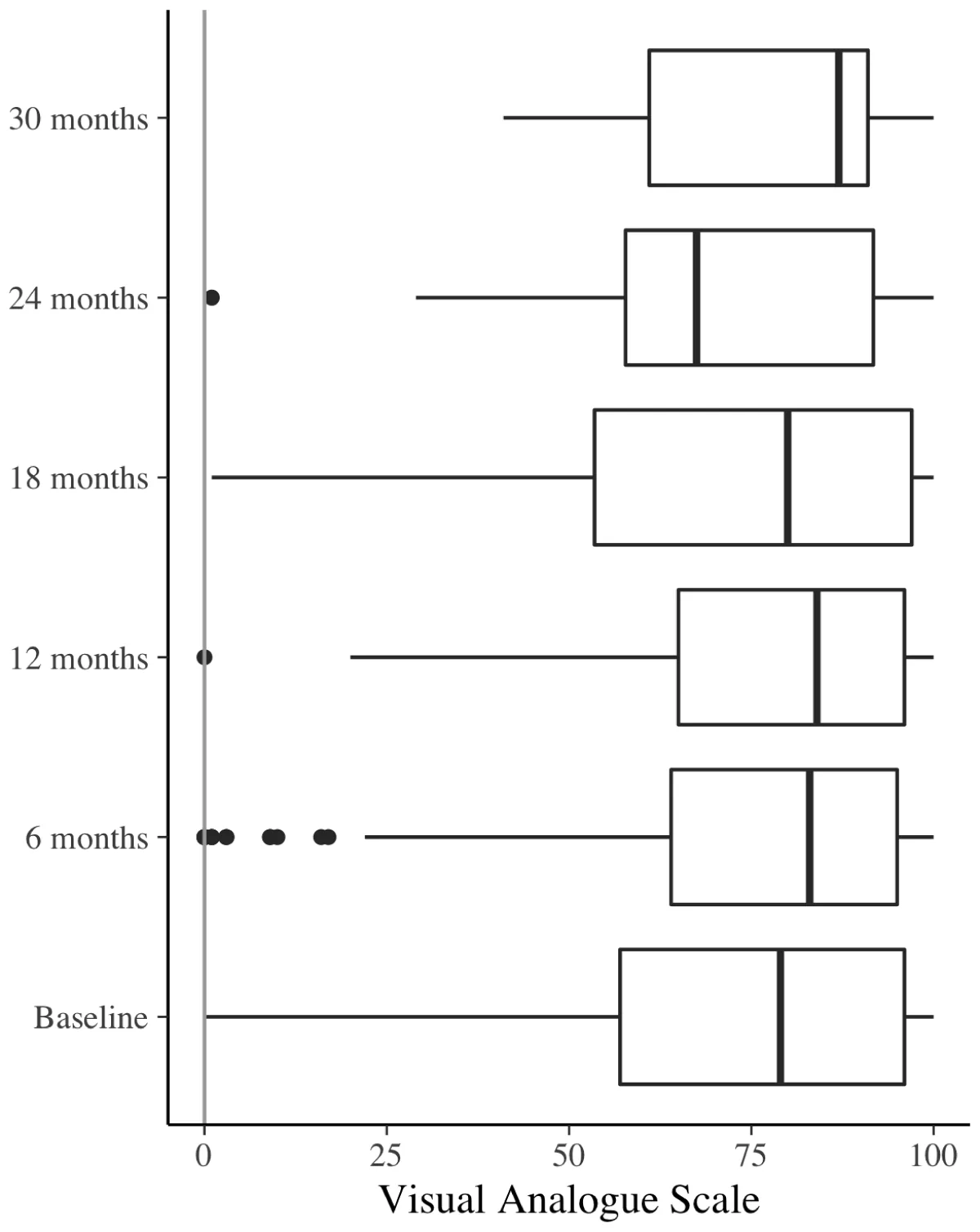
Visual Analogue Scale measuring healthcare experience – medians, quartiles and extreme values at different timepoints.

Figure 2 provides a boxplot showing median, quartiles and extreme values at different timepoints. Median VAS measuring healthcare experience at baseline was 79 and over time median ranged from 69 to 87 with greater variability at later timepoints with smaller respondent numbers. Interquartile range at all time points remained above 52 and below 98. Supplementary Materials Table 3 outlines the number of patients with VAS completed, showing a marked reduction in respondents over time with more than two thirds dropping off from 1019 at baseline and 6 months and a bit over half dropping off at each point thereafter down to 13 at 30 months. It also provides mean and median VAS results at each time point. The random effects model with time as a linear variable assessing trend of VAS over time showed an effect size estimate of 0.2 with 95% credible interval (CI) –0.8 to 1.3 providing no evidence of there being a change over time.

### AQOL4D (Quantitative Analysis)

**Figure 3:**
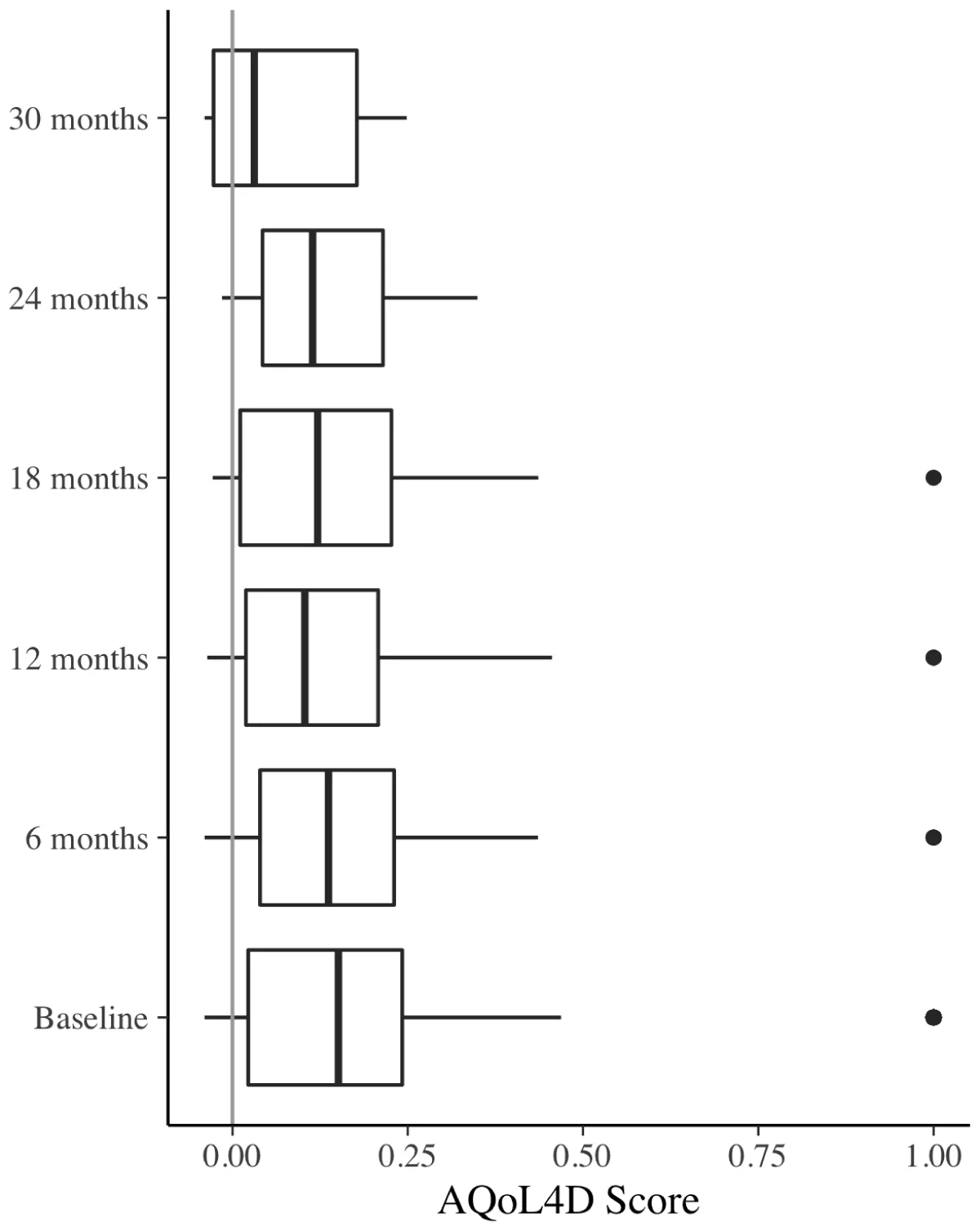
AQoL4D measuring health related quality of life – medians, quartiles and extreme values at different timepoints.

Figure 3 provides a boxplot showing median, quartiles and extreme values at different timepoints. At baseline median AQoL4D was 0.15 and median remained between 0.03 and 0.15 and interquartile range between –0.027 and 0.24 with greatest variability at 30 months with a lower median seen in the small number of respondents. Supplementary Materials Table 4 shows the number of patients with AQoL4D scores as well as mean and median AQoL4D at different timepoints. There were 666 respondents at baseline dropping by a bit over two thirds at 6 months and then a reduction of around half at each subsequent time point down to 11 at 30 months. The random effects model with time as a linear variable assessing trend of AQoL4D over time showed an effect size estimate of –0.007 with 95% CI –0.016 to 0.002 providing no evidence of there being a change over time.

**Figure 4:**
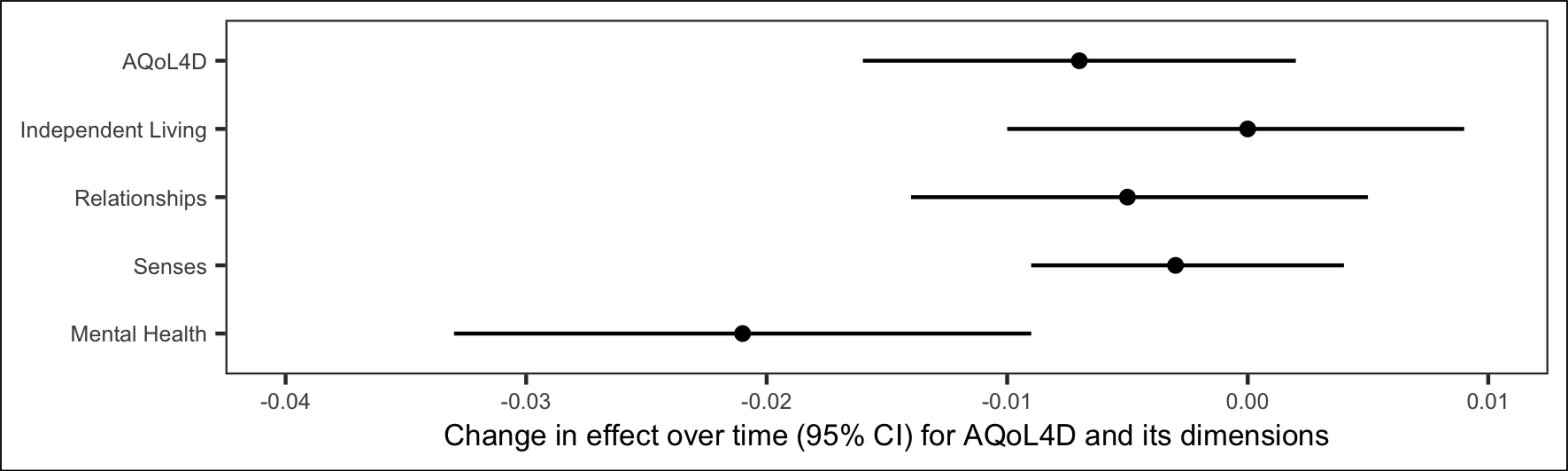
Trend over time for health related quality life measured with AQoL4D and AQoL4D dimensions – effect size estimates.

Figure 4 provides effect size estimates for change over time for AQoL4D measuring health related quality of life and the four different dimensions using a random effects model with time as a linear variable. All estimates included the possibility of a decline or an improvement except for the mental health dimension which showed a small decline over time at –0.021 with 95% CI –0.033 to –0.009. Supplementary Materials Figure 3 provides boxplots with medians, quartiles and extreme values for the four different AQoL4D Dimensions at different timepoints, all of which show greater variability in median and interquartile range at later timepoints which have smaller numbers of respondents.

### Participant drop-out predictions based on AQoL4D (Quantitative Analysis)

Patients with lower AQoL4D scores at baseline were more likely to complete an AQoL4D score at 6 months, with logistic regression showing a significant correlation at –1.2 with 95% CI –2.3 to –0.2. Supplementary Materials Table 5 shows mean AQoL4D score at the preceding timepoint according to presence or absence of the next AQoL4D score.

### Patient survey (Qualitative Analysis)

Validation meetings for thematic analysis took place in: October 2017 at which point there were 147 survey responses for baseline data; February 2018 with 63 responses for 6-month data; August 2019 with 93 responses for 12-month data and 48 responses for 18-month data; December 2019 for 24-month data with 36 responses. The themes that were generated from the three survey questions were home care/local services, access to hospital services, waiting times, coordination of care, communication, consistency of care, healthcare provider qualities, transport/parking and individualisation of care.

Table 2 provides a selection of participant responses to the patient survey questions organised by themes. Theme 1, home care/local services, describes healthcare provided in participants’ homes (such as nurse or GP home visits) or close to home (such as care provided at a local general practice or pharmacy). Theme 2, access to hospital services, refers to participants being able to access care in hospital including emergency department, inpatient and outpatient settings. Theme 3, individualisation of care, relates to care being tailored to each person’s requirements. Theme 4, coordination of care, pertains to healthcare providers working together to care for a patient and sharing relevant information to prevent fragmented care. Theme 5, consistency of care, refers to patients receiving care from the same provider, or from a team who follow a united approach. Theme 6, communication, describes the ways in which healthcare providers provide information and listen to patients. Theme 7, waiting times, includes time spent waiting to receive an appointment, time in the waiting room on the day of an appointment, waiting in the emergency department (or its waiting room) and waiting for health professional review/investigations/procedures when an inpatient. Theme 8, healthcare provider qualities, describes characteristics of doctors, nurses and other health professionals and the way these affect the healthcare experience. Theme 9, transport and parking, relates to how patients get to and from healthcare services including issues relating to proximity of drop off (by private transport/taxi or public transport), parking availability and proximity and expense of all these options.

**Table 2:** Selected participant responses to patient survey organised by theme.


PARTICIPANT NUMBER	TIME	QUESTION	PARTICIPANT RESPONSE TO: QUESTION 1 – *“WHAT WORKS WELL WITH THE HEALTHCARE YOU RECEIVE?”* QUESTION 2 – *“WHAT DOESN’T WORK WELL WITH THE HEALTHCARE YOU RECEIVE?”* QUESTION 3 – *“WHAT COULD BE DONE TO IMPROVE THE HEALTHCARE YOU RECEIVE?”*

			**Theme 1 – Home care/local services**

68	12 months	Question 1	*“It works well for me because nurses come to me at home. The pharmacy deliver the medication. I visit my GP when needed.”*

179	Baseline	Question 2	*“It would be a comfort if I could telephone a health worker for advice if needed.”*

142	Baseline	Question 3	*“Closer facilities for appointments”*

			**Theme 2 – Access to hospital services**

122	6 months	Question 1	*“Critical hospital care, access to medical services”*

124	Baseline	Question 2	*“I need an operation and I cant get into the speacialist to be reviewed*.”

180	18 months	Question 3	*“I think it would be nice to have more often access to the specialists”*

			**Theme 3 – Individualisation of care**

222	Baseline	Question 1	*“An understanding of my needs, appropriate medication. Appropriate healthcare plan.”*

228	6 months	Question 2	*“We have had no problems except for the meals during hospital stay. No real consideration for previous health issue of gastrectomy”*

122	12 months	Question 3	*“Age specific exercise and diet programs (I’m not 80 years old)”*

			**Theme 4 – Coordination of care**

126	12 months	Question 1	*“The availability of a nurse who has my history on hand and ability to contact the local GP and relevant hospital personnel with background knowledge”*

314	12 months	Question 2	*“Because I see so many doctors information from a department to the others is not happening thus the doctors find it hard to treat me.”*

58	Baseline	Question 3	*“Everyone is good except there is overlapping of services & no-one talks to each other. E.g-Xray done already but in hospital they do again.”*

			**Theme 5 – Consistency of care**

203	24 months	Question 1	*“Consistent people who know me & my medical problems”*

10	Baseline	Question 2	*“Seeing GPs who are unfamiliar to me that are unable to make changes or assist”*

196	6 months	Question 3	*“Have one case manager per client”*

			**Theme 6 – Communication**

189	6 months	Question 1	*“All nurses/Gps advising what will happen, why I’m taking the tablets*”

44	Baseline	Question 2	*“Having multiple health professionals at bedside with no introductions & no communication from team to patient.”*

45	Baseline	Question 3	*“Less bombarding with information is needed. Introduction at bedside very important.”*

			**Theme 7 – Waiting times**

179	24 months	Question 1	*“When I needed phone advice it was available immediately.”*

260	12 months	Question 2	*“Going to emergency and waiting for up to 12 hrs or so with uncomfortable chairs”*

151	6 months	Question 3	*“Improve the waiting times in hospital. Recently I waited 2 hours on a ambulance stretcher with 3 ambos waiting with me, what a joke if I moved I could of topped over, they are so narrow.”*

			**Theme 8 – Healthcare provider qualities**

122	Baseline	Question 1	*“All healthcare workers are professional. Caring and positive regarding my care.”*

8	Baseline	Question 2	*“GP lack of understanding and lack of compassion”*

45	24 months	Question 3	*“Patients and understanding.”*

			**Theme 9 – Transport and parking**

309	12 months	Question 1	*“Community transport (link) to various needs. E.g. Hospitals. Specialist- shopping. Visiting family and friends. 1/2 taxi fare, free Ambulance as required. For emergencies.”*

68	24 months	Question 2	*“Well I need transport to take me to appointments and so far I have to depend on taxis or a friend to take me”*

49	6 months	Question 3	*“More parking for disabled at hospitals”*


Supplementary Materials Tables 6 to 8 provide many more examples from the data illustrating the types of comments made by respondents in response to each of the three survey questions. Because saturation was achieved with the number of surveys received at the time of analysis in the validation meetings, surveys received after the meetings were not included in the analysis.

#### Reflexivity (Qualitative Analysis)

##### Personal Reflexivity

Author AA developed the patient survey and led the thematic analysis. Patient commentary on their healthcare experience elucidated during her nursing work may have influenced the question content, and her background in education and qualitative research shaping the formatting of questions in open ended format to maximise breadth of patient response. Authors CN and JJ both work as nephrologists in both inpatient and outpatient settings and as with AA, who has a nursing background, may have viewed patient responses through a healthcare professional lens and may have experienced subconscious defensiveness when encountering criticisms of care provided. Author CN holds executive level roles as clinical services director and head of nephrology unit and like AA whose qualifications in health economics and policy likely viewed responses from an organisation/policy level perspective. Author NL is a researcher who has worked in laboratory, clinical and health services research but has only worked in a healthcare environment from a research perspective, she therefore may have experienced minimal subconscious defensiveness when reviewing patient criticism of healthcare.

##### Interpersonal Reflexivity

Participants were given the surveys by study staff, and concerns about staff knowing about criticisms of their healthcare may have affected their responses. Participants may have not felt comfortable fully disclosing negative experiences and may have been more likely to report positive experiences because of this power dynamic.

##### Methodological Reflexivity

This study was conducted using an inductive approach in which survey responses were coded and themes developed based on patients’ written answers to open ended questions. It is possible that the authors’ research and clinical experience and exposure to existing literature may have led to their having preconceived ideas about what themes may emerge. However, researchers did not try to fit pre-defined themes to the survey responses and sought to follow an inductive approach as closely as possible.

##### Contextual Reflexivity

This study was conducted in both a hospital and community environment with participants being people with chronic and complex care needs. Participants may at times not have had the energy or felt they were well enough to provide detailed responses and at times any response at all.

## Discussion

This real-world prospective mixed-methods observational cohort study conducted as an implementation evaluation study describes the experiences of patients with chronic illness in Victoria, Australia, receiving an integrated care model seeking to help patients stay well at home after hospital discharge. We found that patients who received the Western HealthLinks program maintained their HRQoL and satisfaction with their healthcare, with no significant change to AQoL4D or VAS measuring healthcare satisfaction over time. Thematic analysis identified several strong themes which highlighted that patients value local, timely, individualised and coordinated care that is easy to access with practitioners who communicate clearly, highlighting that patients value integrated care.

Median AQoL4D at baseline was very low at 0.15, far below the national average of 0.81, measured from a sample of the Australian population in 2007 [16], illustrating the poor HRQoL that patients with chronic and complex care needs participating in Western HealthLinks were experiencing. The study population were living with chronic illness and were at risk of progression of illness and decline in their health and HRQoL. Therefore, maintenance of HRQoL as assessed using the AQoL4D, with no clinically meaningful decline over time, suggests a possible beneficial effect of the Western HealthLinks program on HRQoL. The lower limit of the 95% CI represents the largest potential decline that is not rejected with alpha at 0.05 when modelling the data with a linear mixed model including random intercepts. For all measured endpoints, these worst-case estimates are sufficiently small so as to be clinically acceptable. For four out of five of the measured outcomes (AQoL4D and all dimensions except Mental Health) an improvement over time is also plausible. It is worth noting the substantial mental health challenges faced by patients with chronic illness and considering additional psychological support and mental health input in future programs supporting this patient population.

An umbrella review of systematic reviews addressing integrated care-based interventions for people with chronic disease [10] found 18 of 41 included reviews with a benefit to QoL, however much of this data related to interventions targeting single conditions, with only three included reviews addressing chronic disease in general [17,18,19]. Two of these three reviews [17,18] found improved QoL and one [19] mixed results. There are mixed findings from Australian studies using integrated care-based interventions for people with chronic disease, with two randomised controlled trials finding improvements to QoL [20,21], one randomised controlled trial finding no improvement [8] and one quasi-experimental controlled trial finding no improvement [22]. There is great variety in intervention and study design as well as differences in study populations likely contributing to the heterogeneous findings across studies.

Median VAS measuring healthcare satisfaction at baseline was quite high at 79 and following the implementation of Western HealthLinks, there was no meaningful decline over time with an improvement over time being plausible. As there was no control group, it is not possible to know if patients would have had a notable difference to their AQoL4D or healthcare satisfaction if they had received usual care. However, the qualitative analysis provided insights into the role of Western HealthLinks in patient healthcare satisfaction.

An umbrella review investigating integrated care-based interventions for people with chronic disease found an improvement in patient satisfaction for seven of the ten included randomised controlled trials [23]. However, no Australian studies of integrated care-based interventions, targeting chronic disease in general, with quantitative assessment of healthcare satisfaction were identified.

There were nine main themes that emerged from the thematic analysis. The strongest theme over all was home care/local services: Patients appreciated the home care/local services that they had, but wanted more of it. The other themes were access to hospital services, waiting times (especially in the emergency department), coordination of care, communication, consistency of care, healthcare provider qualities, transport/parking and individualisation of care.

The issue of provision of home care/local services illustrates the toll of the chronic nature of illness on patients, especially if requiring very frequent contact with health providers. Patients appreciated the care they received at home as part of Western HealthLinks including home visits and phone support. Provision of care in the home or close to home can save time and energy expended from their already depleted reserves. Likewise, when patients need to come to hospital, they wanted to come to the right person and place and avoid long periods in a waiting room. There was a strong preference to have someone to coordinate events, and they valued the care coordination provided by Western HealthLinks. Patients with chronic illness may suffer from attentional fatigue thereby compromising their ability to plan and organise, making coordinated care very important. These findings are consistent with a content analysis of patient survey data by another team of researchers, which identified that continuity and coordination were highly rated and valued domains [24].

Individualisation of care was another theme that emerged. Patients wished to be listened to and have providers understand their specific priorities. Patients wanted care tailored to their needs, expectations and level of health literacy. Prompt and reliable follow up was also highly valued by patients. Patients appreciated friendly, kind and patient focused staff that listened and did not make assumptions about them. They wanted staff to work with them rather than having a paternalistic approach. This is supported by data from a systematic review of patient complaint data where 27.5% of complaints resulted from staff attitudes and behaviour [25]. It is important to be mindful when using this partnership approach, that some patients may not want to make decisions or may too unwell to participate [26].

In summary, the thematic analysis presented a consistent pattern with the key themes being replicated at each time point. These patients wanted to receive care convenient to where they live and when needing access to hospital services, didn’t want to wait. They wanted care that was individualised, coordinated and well communicated to themselves and amongst the various health providers. Patients wanted systems and structures that decrease the overall burden that chronic illness places on them. It highlighted that patients wanted “person centred coordinated care”, the National Voices definition of integrated care [4].

There were lower numbers of completed AQoL4D, VAS and healthcare satisfaction survey at later timepoints. In part, this can be explained by there being fewer patients in the study who had been enrolled for long enough to provide data at later timepoints in addition to the substantial number of patients who died over the course of the study. However, it is worth noting that patients with higher AQoL4D scores at a given timepoint were less likely to answer the AQoL4D questionnaire at the next timepoint. A potential reason for this could be that those with lower HRQoL are particularly motivated to provide feedback regarding their wellbeing and healthcare experience. Quantitative analysis of other patient outcomes (including hospital admissions/length of stay data) will be published in a separate outcome paper looking at all patients in the program including those who did not complete patient experience surveys.

Western HealthLinks finished in November 2019 and after its formal evaluation, taking into account this qualitative patient feedback, it was internalised as business as usual. The decision to internalise the program was made because, in addition to the findings outlined in this evaluation, Western HealthLinks was associated with reduced hospital readmissions (with a formal evaluation of healthcare utilisation data to be discussed in a separate publication), thereby reducing pressure on the healthcare service. The program was expanded to include COVID monitoring during the pandemic. Since transitioning the program internally with no external support from SilverChain due to funding limitations, patient-reported experience measures and patient-reported outcome measures continue to be used to assess aspects of the programs that have stemmed from this initial program, however results are not directly comparable to this evaluation. Patients and carers continue to participate in the Western Health Chronic Disease Alliance steering group, Safe Care, Best Care and Quality and Safety meetings.

Study strengths included the value of analysing real-world data offering insight into the experiences of patients receiving care through a government funded service rather than highly selected patients enrolled in a clinical trial. Additionally, this study included patients with a multitude of different chronic illnesses, rather than focusing on just one particular condition. The catchment for Western Health includes multiple areas of relative socio-economic disadvantage as well as some more privileged areas, and includes a culturally and linguistically diverse population, making the results of this study likely to be more generalisable than studies conducted in more homogenous populations. However, patients who required an interpreter to complete the assessments were not eligible to participate, limiting the generalisability to only patients who were comfortable using the English language. The mixed methods approach enables quantitative measurement of patients’ experiences using VAS, a simple measurement tool, as well as a more sophisticated, highly validated measure of HRQoL, the AQoL4D tool as well as the rich qualitative data outlined in the thematic analysis.

A major limitation of this study is the substantial number of patients who did not complete surveys/questionnaires at later time points which introduces the risk of attrition bias. Another weakness is the absence of a control group, so there is no data on what would have happened to HRQoL in patients eligible for Western HealthLinks who got usual care rather than the Western HealthLinks program, and those who did not wish to participate were not a comparable group. Future studies incorporating multiple health care networks and the inclusion of control groups would help to address these issues.

## Conclusion

This study describes patients’ experiences of an integrated care model supporting patients with chronic disease post hospital discharge with a view to helping patients stay well at home in Victoria, Australia. Patients are at risk of fragmented care when they transition from a hospital stay to home which can negatively impact their healthcare. The Western HealthLinks integrated care chronic illness management program sought to address this issue for patients with chronic illness to support them upon hospital discharge with a view to preventing hospital readmission. This real-world prospective mixed-methods observational cohort study conducted as an implementation evaluation assessed patients’ experiences of the Western HealthLinks program and found patients in the program maintained their HRQoL and satisfaction with their healthcare over time. Qualitative data showed patients’ appreciation for the home-based care and care coordination as part of Western HealthLinks. The thematic analysis identified strong themes highlighting that patients wanted individualised, coordinated, timely, consistent care with high quality healthcare providers with good communication, access to both local/home-based and hospital care with easy transport/parking options at the hospital. Key learnings from this project were that people living with chronic disease who are at high risk of hospital readmission experience very low levels of quality of life and greatly value local/home-based healthcare given the challenges they experience when receiving care further afield. This is a critical component that should be incorporated when designing models of care for this population. Future studies comprising multiple health care networks with control groups would be valuable. Despite its limitations, this study has shown that an integrated care chronic illness management program assisting patients with their healthcare after discharge from hospital to help them stay well at home may help to maintain patient HRQoL and healthcare satisfaction. The qualitative component of this study reinforced that patients with chronic illness identified integrated care as a vital part of their healthcare, underscoring the importance of designing healthcare models with an integrated care design to better meet patient requirements.

## Additional File

The additional file for this article can be found as follows:

10.5334/ijic.8980.s1 Supplementary Materials. Supplementary Materials tables 1 to 8; figures 1 to 3 and forms 1 to 2.
