# Supplementary material for: Patients’ Experiences of the Western HealthLinks Integrated Care Chronic Illness Management Program: A Prospective Mixed-Methods Observational Cohort Study
Source: Int J Integr Care. 2026 Sep 7;26(3):14. doi: 10.5334/ijic.8980 (PMC13565075; doi:10.5334/ijic.8980)
Supplement: Supplementary file 1 [file ijic-26-3-8980-s1.pdf]

Supplementary Materials

Patients' experiences of the Western HealthLinks integrated care chronic illness management program: a prospective mixed-methods observational cohort study

Supplementary Materials Table 1: Questions used to assess risk of readmission

Debility, Psychosocial Risk Score		
**Question**	**Available Score**	**Patient Score**
**1.** Does the patient experience difficulty walking (e.g. unable to walk 5 metres in 5 seconds), or had a slip, trip, or fall in the past 6 months?	1	
**2.** Does the patient have memory problems or confusion?	1	
**3.** Is the patient being treated for anxiety, depression or other mental illness?	1	
**4.** Will the patient experience any homelessness for the month after they leave the hospital?	1	
**5.** Does the patient have inadequate food available in their home?	1	
**6.** Does the patient have inadequate heating and cooling in their home?	1	
**7.** Will the patient experience difficulty caring for themselves or have inadequate carer support for the 30 days after they leave hospital?	1	
**8.** Does the patient or carer believe the patient might unexpectedly return back to a hospital bed in the 30 days after they leave the hospital?	1	
**9.** Do you (Navigator) believe the patient might unexpectedly return back to a hospital bed in the 30 days after the patient leaves the hospital?	1	
**Total Debility, Psychosocial Risk Score**	9	

Supplementary Materials Table 2: Readmission risk matrix

Debility, Psychosocial Score (Readmission Risk)	
**High** (Total score ≥ 3 or any points scored for question 8 or 9)	(red)
**Medium** (Total score 1-2 and no points scored for question 8 or 9)	(amber)
**Low** (Total score 0)	(green)

Supplementary Materials Figure 1: Flowchart outlining process for determining Western HealthLinks eligibility and eligibility to participate in patient experience study

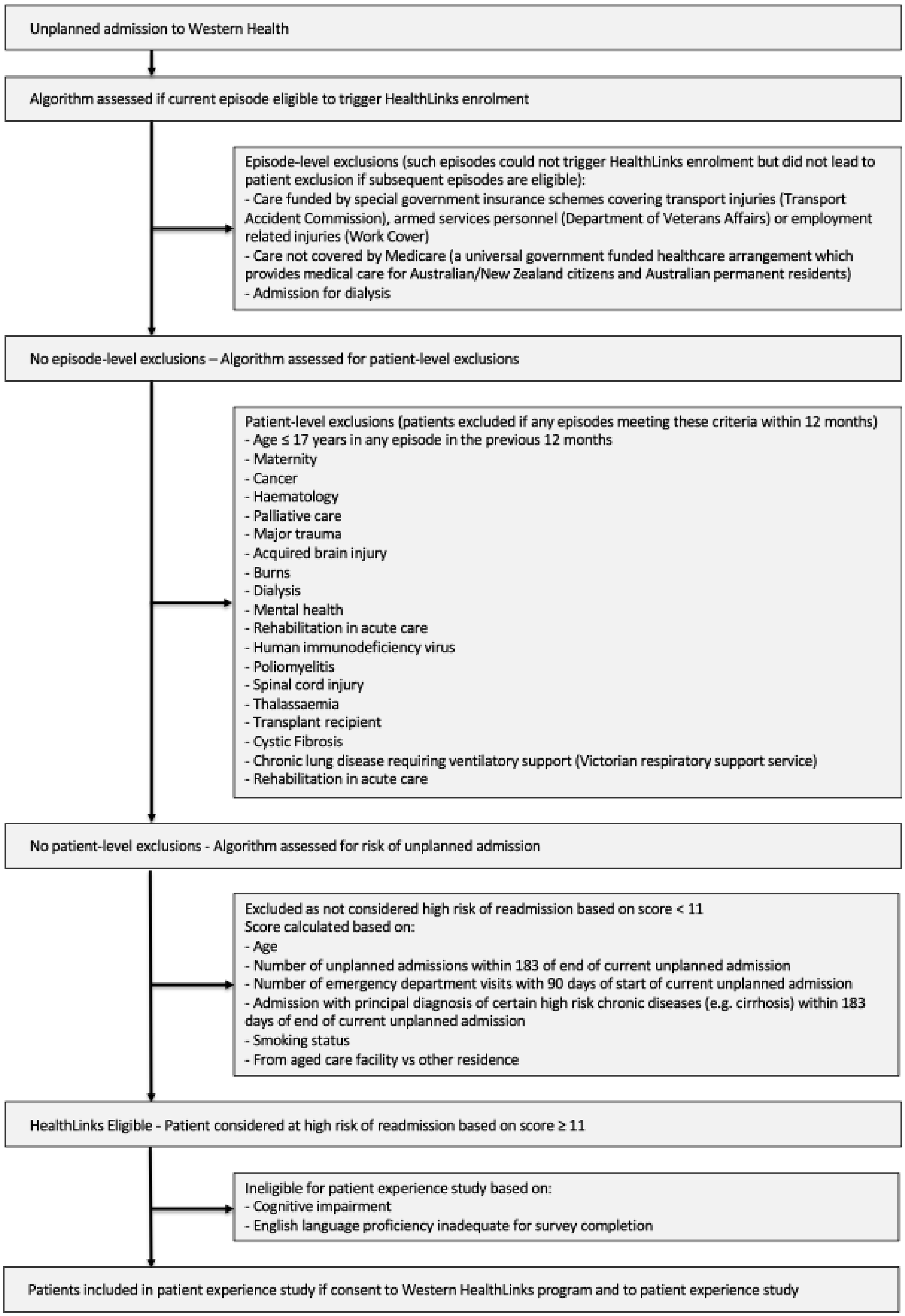

Supplementary Materials Figure 2: Components of Western HealthLinks Intervention

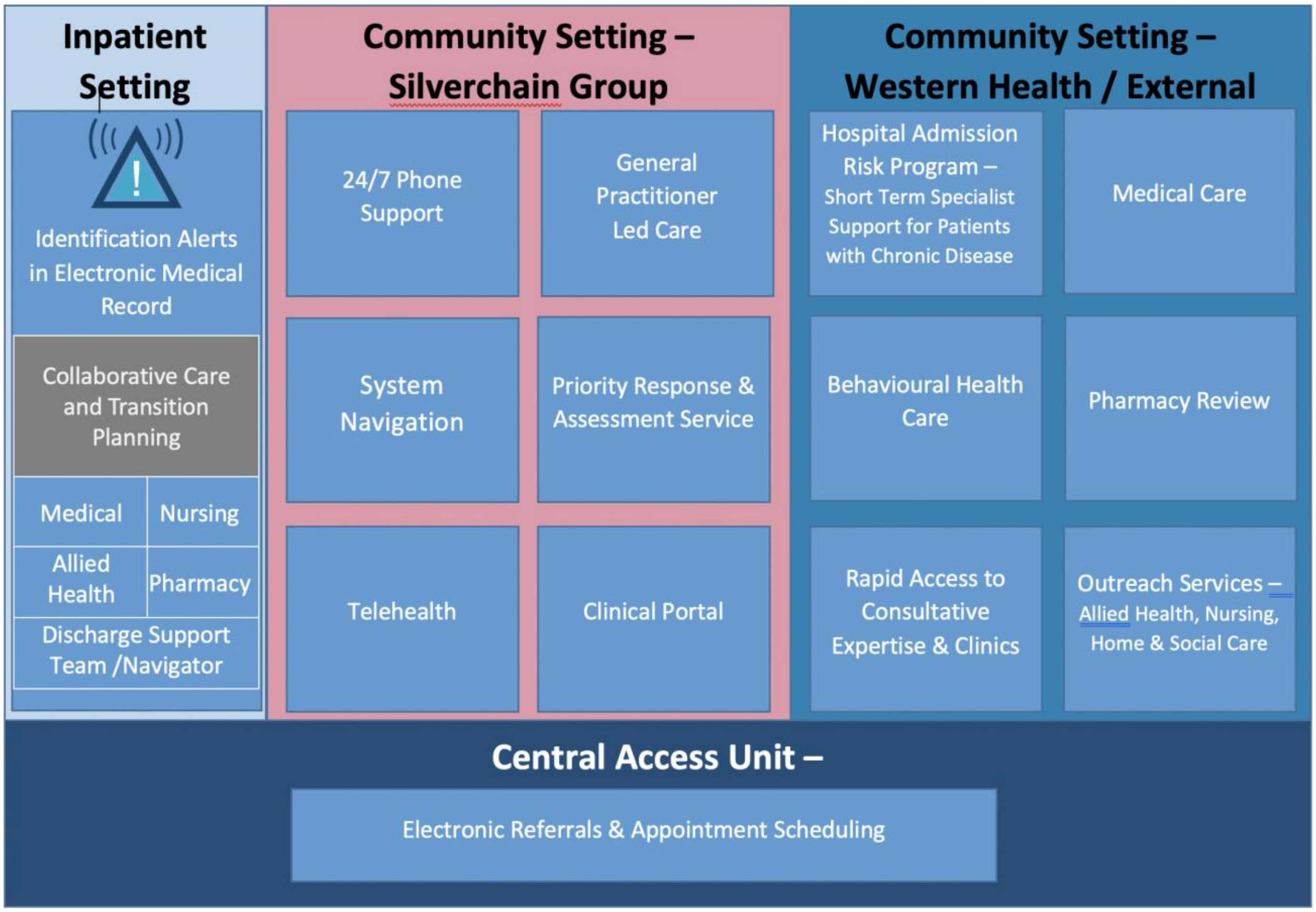

Supplementary Materials Form 1: Participant Information and Consent Form

PATIENT LABEL

Booklet

WHCOR26

Western Health

Western Health Low Risk Human Research Ethics Panel

Participant Information and Consent Form

Version Two, Dated 5th October, 2016
Sunshine Hospital

Full Project Title: Western Health Healthlinks – An Evaluation

Principal Researcher: Associate Professor Craig Nelson – Western Health

Professor Geoff McColl – Melbourne Health

Associate Researcher(s): Dr Ann Allenby – Western Health

Jason Plant – Western Health

Lebe Malkoun – Western Health

Andrew Edwards - Silverchain

This Participant Information and Consent Form is 6 *pages* long. Please make sure you have all the pages.

1. Your Consent
You are invited to take part in this evaluation project.

This Participant Information contains detailed information about the evaluation. Its purpose is to explain to you as openly and clearly as possible all the procedures involved in this project before you decide whether or not to take part in it.

Please read this Participant Information carefully. Feel free to ask questions about any information in the document. You may also wish to discuss the evaluation with a relative or friend or your local health worker. Feel free to do this.

Once you understand what the evaluation is about and if you agree to take part in it, you will be asked to sign the Consent Form. By signing the Consent Form, you indicate that you understand the information and that you give your consent to participate in the evaluation.

You will be given a copy of the Participant Information and Consent Form to keep as a record.

2. Purpose and Background

There are two main reasons for this evaluation:

- To get feedback from you about the program so we can use this information to further develop the program so it meets patient needs, and
- We are interested in seeing if the Healthlinks program improves your quality of life and satisfaction with the healthcare system

A maximum number of 5000 people will participate in this project.

Previous experience has shown that providing an extra level of care and support to patients who have chronic and complex conditions helps improve the patients overall state of health and satisfaction with healthcare. Research has also shown that programs such as Healthlinks help people feel more confident and secure at home and helps avoid crisis admissions to hospital. It is important that in starting a new program like this that we evaluate how useful it is from a patient's point of view and to see if it does improve quality of life and satisfaction.

You are invited to participate in this evaluation because you have agreed to be enrolled in the Healthlinks program.

3. Procedures

Participation in this project will involve

- Completing a survey that will ask you about your quality of life (using a validated tool) and your satisfaction with the healthcare system which should take no more than 10 minutes
- We ask that this survey is done when you first enrol on Healthlinks and every six months until the end of the project (at the latest October 2020)
- You will be asked to do your survey at the same time a Healthlinks staff member visits you at home or by phone during a phone support conversation
- You can feel free to withdraw at any time – just let the Healthlinks staff know
- If you go on to develop a condition that requires frequent stays in hospital such a dialysis or cancer treatment you will be withdrawn from the evaluation – we will let know if this happens

4. Possible Benefits

We cannot guarantee or promise that you will receive any benefits from this evaluation, however, your feedback will help us improve the program from which you may directly benefit.

5. Possible Risks

We do not anticipate there are any risks to you from taking part in this evaluation.

6. Alternatives to Participation

If you do not want to participate in the evaluation you will continue to receive care from the Healthlinks program.

If at any time you have feedback about the program and how it might be improved please feel free to let a Healthlinks or any Western Health staff member know.

Alternatively feel free to contact Jason Plant the Healthlinks Director on 03 8345 6666.

7. Privacy, Confidentiality and Disclosure of Information
Any information obtained in connection with this evaluation and that can identify you will remain confidential. There are strict rules around how information is stored and secured to protect your privacy. If you give us your permission by signing the Consent Form, we plan to share the results of the evaluation with Department of Health and Human Services and other interested health services.

In any publication, information will be grouped together in a way that you cannot be identified. If we want to use a quote that you have made in a publication we will not identify you.

In accordance with relevant Australian and/or Victorian privacy and other relevant laws you have the right to access the information collected and stored by the researchers about you. You also have the right to request that any information with which you disagree be corrected. Please contact one of the researchers named below if you would like to access your information.

8. New Information Arising During the Project
During the evaluation, new information about the risks and benefits of the project may become known to the researchers. If this occurs, you will be told about this new information. This new information may mean that you can no longer participate in this research. If this occurs, the person(s) supervising the research will stop your participation. This will not affect your participation in Healthlinks.

9. Results of evaluation
The results of the evaluation will be made available in report at the end of an evaluation. If you wish to have a copy of this report or a summary please contact the research office on (03) 83457245

10. Further Information or Any Problems
If you require further information or if you have any problems concerning this evaluation, you can contact the principal researcher via the research office. The researchers responsible for this project are Associate Professor Craig Nelson and Professor Geoff McColl, a message can be left with the research office on (03) 83457245 and you will contacted as soon as practical.

11. Other Issues
If you have any complaints about any aspect of the project, the way it is being conducted or any questions about your rights as a research participant, then you may contact:

Position:	Manager, Western Health Office for Research
Telephone:	(03) 8395 8073
Email:	ethics@wh.org.au

(You will need to tell the Manager the name of one of the researchers given in section 10 above.)

12. Participation is Voluntary

Participation in any evaluation is voluntary. If you do not wish to take part you are not obliged to. If you decide to take part and later change your mind, you are free to withdraw from the project at any stage.

Your decision whether to take part or not to take part, or to take part and then withdraw, will not affect your routine treatment, your relationship with those treating you or your relationship with Western Health.

Before you make your decision, a member of the research team will be available to answer any questions you have about the research project. You can ask for any information you want. Sign the Consent Form only after you have had a chance to ask your questions and have received satisfactory answers.

If you decide to withdraw from this project, please notify a member of the research team before you withdraw. This notice will allow that person or the research supervisor to inform you if there are any health risks or special requirements linked to withdrawing.

13. Ethical Guidelines

This evaluation will be carried out according to the *National Statement on Ethical Conduct in Human Research* (2007) produced by the National Health and Medical Research Council of Australia. This statement has been developed to protect the interests of people who agree to participate in human research studies.

The ethical aspects of this research project have been approved by the Western Health Low Risk Human Research Ethics Panel.

14. Reimbursement for your costs

Your participation is voluntary and you will not be paid for your participation in this project.

15. Consent Form

Version 1 Dated 30th August 2016
Site: Sunshine Hospital
Project title: Western Health Healthlinks – An Evaluation

I have read, or have had read to me in my first language and I understand the Participant Information version 1 dated 24th August 2016.

I freely agree to participate in this project according to the conditions in the Participant Information.

I will be given a copy of the Participant Information and Consent Form to keep

The researcher has agreed not to reveal my identity and personal details if information about this project is published or presented in any public form.

Participant's Name (printed) ……………………………………………………

Signature………………………………… Date

Name of Witness to Participant's Signature (printed) …………………………………

Signature………………………………… Date

Declaration by researcher*: I have given a verbal explanation of the research project, its procedures and risks and I believe that the participant has understood that explanation.

Researcher's Name (printed) ……………………………………………………

Signature………………………………… Date

* A senior member of the research team must provide the explanation and provision of information concerning the research project.

Note: All parties signing the Consent Form must date their own signature.

Western Health
Western Health

WITHDRAWL OF CONSENT FORM

Full Project Title: Western Health Healthlinks – An Evaluation

I hereby wish to WITHDRAW my consent to participate in the evaluation described above and understand that such withdrawal WILL NOT jeopardise any treatment or my relationship with Western Health.

☐ I am happy for the information collected up to this point to be used in the evaluation

☐ I would all the information collected up to this point to be removed from the evaluation

Participant's Name (printed) ……………………………………………………

Signature………………………………… Date

Supplementary Materials Form 2

Western Health

PATIENT LABEL

Western Healthlinks – An Evaluation

Please take 5-10 minutes to complete the following questions

Part 1 – your feedback

The word healthcare is used to describe the whole health care system such as hospital, nurses who might visit you at home, pharmacy, GP, etc.

1. What works well with the healthcare you receive?

2. What doesn't work well with the healthcare you receive?

3. What could be done to improve the health care you receive?

4. How satisfied are you with your healthcare (put a mark or cross anywhere on the line that best suits your answer)

Not satisfied ____ Highly satisfied

Part 2 – your quality of life

The following questions were developed by Monash University and they are designed to measure your quality of life. Please tick the box next to the response that best fits your situation.

1. Do you need any help looking after yourself? (For example: dressing, bathing, eating)

☐ I need no help at all

☐ Occasionally I need some help with personal care tasks

☐ I need help with the more difficult personal care tasks

☐ I need daily help with most or all personal care tasks

2. When doing household tasks: (For example: cooking, cleaning the house, washing)

☐ I need no help at all

☐ Occasionally I need some help with household tasks

☐ I need help with the more difficult household tasks

☐ I need daily help with most or all household tasks

3. Thinking about how easily you can get around your home and community:

☐ I get around my home and community by myself without any difficulty

☐ I find it difficult to get around my home and community by myself

☐ I cannot get around the community by myself, but I can get around my home with some difficulty

☐ I cannot get around either the community or my home by myself

4. Because of your health, your relationships (for example: with your friends, partner or parents) generally:

☐ Are very close and warm

☐ Are sometimes close and warm

☐ Are seldom close and warm

☐ I have no close and warm relationships

5. Thinking about your relationship with other people:

☐ I have plenty of friends, and am never lonely

☐ Although I have friends, I am occasionally lonely

☐ I have some friends, but am often lonely for company

☐ I am socially isolated and feel lonely

6. Thinking about your health and your relationship with your family:

☐ My role in the family is unaffected by my health

☐ There are some parts of my family role I cannot carry out

☐ There are many parts of my family role I cannot carry out

☐ I cannot carry out any part of my family role

7. Thinking about your vision, including when using your glasses or contact lenses if needed:

☐ I see normally

☐ I have some difficulty focusing on things, or I do not see them sharply. For example: small print, a newspaper or seeing objects in the distance

☐ I have a lot of difficulty seeing things. My vision is blurred. For example: I can see just enough to get by with

☐ I only see general shapes, or am blind. For example: I need a guide to move around

8. Thinking about your hearing, including using your hearing aid if needed:

☐ I hear normally

☐ I have some difficulty hearing or I do not hear clearly. For example: I ask people to speak up, or turn up the TV or radio volume

☐ I have difficulty hearing things clearly. For example: Often I do not understand what is said. I usually do not take part in conversations because I cannot hear what is said

☐ I hear very little indeed. For example: I cannot fully understand loud voices speaking directly to me

9. When you communicate with others: (For example: by talking, listening, writing or signing.)

☐ I have no trouble speaking to them or understanding what they are saying

☐ I have some difficulty being understood by people who do not know me. I have no trouble understanding what others are saying to me

☐ I am only understood by people who know me well. I have great trouble understanding what others are saying to me

☐ I cannot adequately communicate with others

10. Thinking about how you sleep:

☐ I am able to sleep without difficulty most of the time

☐ My sleep is interrupted some of the time, but I am usually able to go back to sleep without difficulty

☐ My sleep is interrupted most nights, but I am usually able to go back to sleep without difficulty

☐ I sleep in short bursts only. I am awake most of the night

11. Thinking about how you generally feel:

☐ I do not feel anxious, worried or depressed

☐ I am slightly anxious, worried or depressed

☐ I feel moderately anxious, worried or depressed

☐ I am extremely anxious, worried or depressed

12. How much pain or discomfort do you experience:

☐ None at all

☐ I have moderate pain

☐ I suffer from severe pain

☐ I suffer unbearable pain

Thank you for your time taken to fill out these questions. Your feedback will help us make changes to the Healthlinks program.

© AQoL-4D, Centre for Health Economics, Monash University

Supplementary Materials Table 3: Mean and median Visual Analogue Scale (0-100) indicating patient satisfaction with healthcare

Time	Number of patients with VAS completed	Mean	Median
Baseline	1019	73	79
6 Months	327	75	83
12 Months	160	77	84
18 Months	76	74	80
24 months	36	72	69
30 months	13	79	87

Supplementary Materials Table 4: Mean and median AQoL4D at different timepoints

Time	Number of patients with AQoL4D completed	Mean	Median
Baseline	666	0.18	0.15
6 Months	217	0.15	0.14
12 Months	104	0.11	0.1
18 Months	53	0.14	0.12
24 months	22	0.13	0.11
30 months	11	0.07	0.03

Supplementary Materials Figure 3: AQoL4D Dimensions – medians, quartiles and extreme values at different timepoints

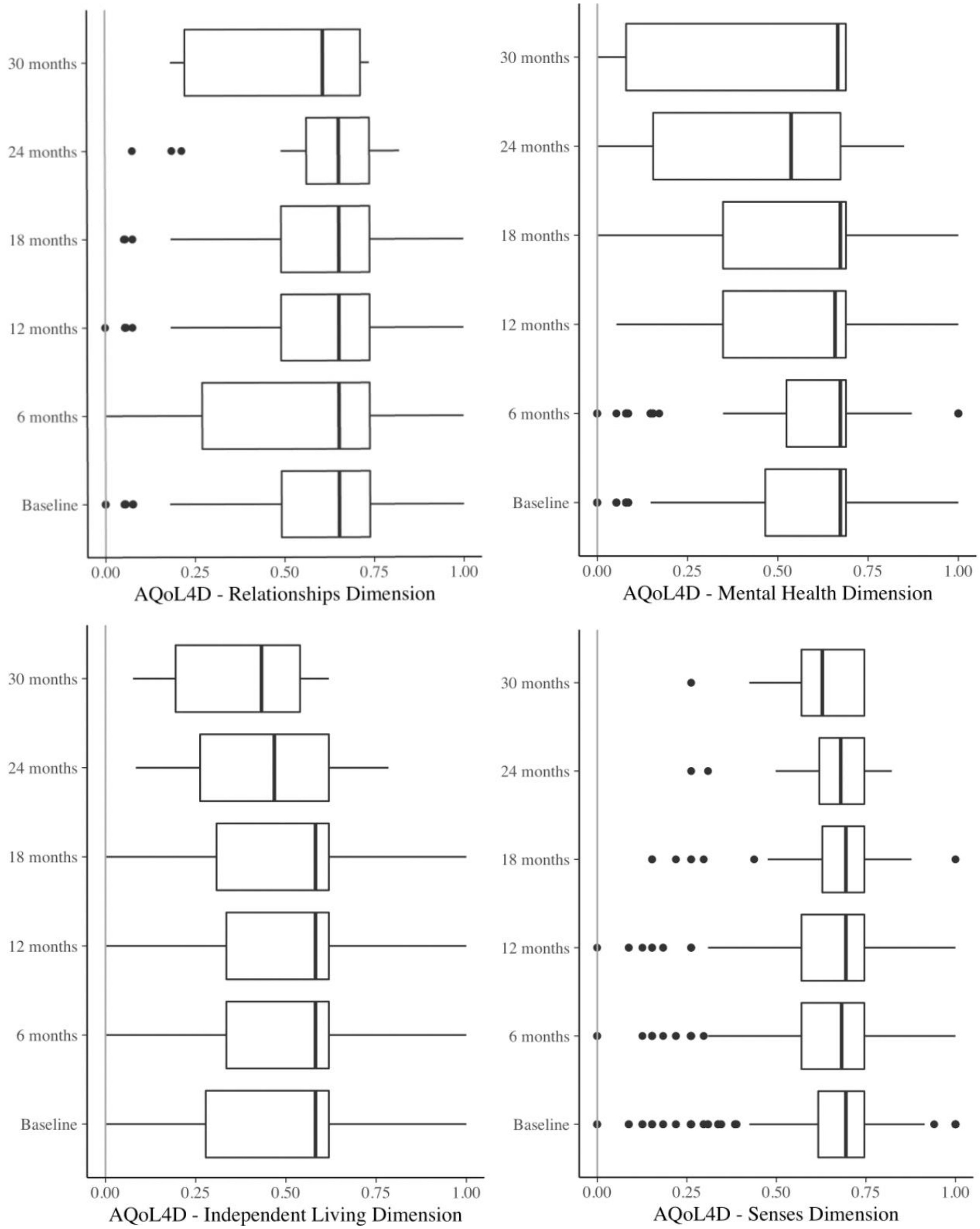

Supplementary Materials Table 5: Mean AQoL4D scores at earlier timepoints according to presence/absence of subsequent score

Time	Mean AQoL4D when next score present	Mean AQoL4D when next score absent
AQoL4D score at baseline	0.15 (when 6-month score present)	0.19 (when 6-month score absent)
AQoL4D score at 6 months	0.14 (when 12-month score present)	0.15 (when 12-month score absent)
AQoL4D score at 12 months	0.14 (when 18-month score present)	0.10 (when 18-month score absent)
AQoL4D score at 18 months	0.1 (when 24-month score present)	0.15 (when 24-month score absent)
AQoL4D score at 24 months	0.09 (when 30-month score present)	0.15 (when 30-month score absent)

Supplementary Materials Table 6: Comments made in response to question 1 – "What works well with the healthcare you receive?"

Themes	Responses to question 1 "What works well with the healthcare you receive?"
Home care/local services	"Having a good GP who does home visits." – participant 104, baseline<br>"The knowledge that I receive regular phone calls to check there are no further problems with my health" – participant 202, 6 months<br>"Home delivery from pharmacy." – participant 21, 6 months<br>"Home doctor service when GP closed." – participant 49, 6 months<br>"The services provided by nurses, especially the home visits." – participant 35, 12 months<br>"It works well for me because nurses come to me at home. The pharmacy deliver the medication. I visit my GP when needed." – participant 68, 12 months<br>"The people (Dr Nurses etc) + Silver Chain who ring me" – participant 18, 18 months<br>"I receive good care from my GP + also telephone support from Western HealthLinks." – participant 59, 24 months<br>"Excellent GP care" – participant 184, 24 months
Access to hospital services	"being able to access specialist renal teams when required." – participant 10, baseline<br>"Specialist vascular service works well" – participant 17, baseline<br>"access to the hospital" – participant 32, baseline<br>"critical hospital care, access to medical services" – participant 122, 6 months<br>"appointments at clinics with specialists" – participant 96, 12 months<br>"I get to see endo-renal on regular visits" – participant 345, 12 months<br>"Hospital – good care" – participant 49, 18 months<br>"I have been happy with the treatment I received when I was in hospital" – participant 114, 24 months
Individualis-ation of care	"an understanding of my needs, appropriate medication. Appropriate healthcare plan." – participant 222 baseline<br>"chemist that know my medical requirements well" – participant 19, 6 months<br>"My ASMA Plan" – participant 22, 12 months<br>"ME – an awareness of my problems + what to do" – participant 134, 18 months<br>"I'm treated as thought I'm the only one" – participant 146, 18 months
Coordination of care	"When specialist and GP liaise and where I am in hospital and can still be involved in my care." – participant 11, baseline<br>"Having a good GP to coordinate health problems." – participant 49, baseline<br>"Regular contact with navigators with ideas on how to approach problems / Hurdles." – participant 40, 6 months<br>"Co-ordination between GP and Specialists (Heart, Kidneys and Arthritis)" – participant 213, 6 months<br>"The availability of a nurse who has my history on hand and ability to contact the local GP and relevant hospital personnel with background knowledge" – participant 126, 12 months<br>"I appreciate how all my healthcare providers are looking in conjunction with each other to provide the best care for my physical + mental health." – participant 275, 12 months<br>"Doctor's + hospital working together" –participant 227, 24 months
Consistency of care	"having regular healthcare providers" – participant 29, baseline<br>"Staying with the one doctor, as she knows my history." – participant 117, baseline<br>"A secure relationship with healthcare workers, consistency of healthcare" – participant 222, baseline<br>"After my discharge from hospital the support was invaluable" – participant 196, 12 months<br>"Consistent people who know me & my medical problems" – participant 203, 24 months
Communic-	"Being provided with enough information to make informed decisions." – participant 204, baseline

ation	“All nurses/Gps advising what will happen, why I'm taking the tablets” – participant 189, 6 months<br>“explained everything so I understood” – participant 193, 6 months<br>“I receive good advice” – participant 29, 24 months
Waiting times	“The hospital emergency department are usually quick in addressing my pain.” – participant 64, baseline<br>“I get appointments easily when I need it.” – participant 89, baseline<br>“I am always seen at emergency at sunshine hospital straight away.” – participant 126, baseline<br>“When I needed phone advice it was available immediately.” – participant 179, 24 months
Healthcare provider qualities	“The ambulance, doctors and nurses very kind” – participant 2, baseline<br>“All healthcare workers are professional. Caring and positive regarding my care.” – participant 122, baseline<br>“GP has compassion, listens to my problems is very pleasant.” – participant 31, 6 months<br>“Concientous + informative” – participant 162, 6 months<br>“Staff have been friendly and helpful in all aspects of my healthcare” – participant 228, 6 months<br>“The staff were friendly and helped me through a lot of tough times.” – participant 143, 12 months<br>“The way they treat you they all nice” – participant 225, 18 months
Transport/ parking	“Community transport (link) to various needs. E.g. Hospitals. Specialist- shopping. Visiting family and friends. 1/2 taxi fare, free Ambulance as required. For emergencies.” – participant 309, 12 months

Supplementary Materials Table 7: Comments made in response to question 2 – “What doesn’t work well with the healthcare you receive?”

Themes	Responses to question 2 “What doesn’t work well with the healthcare you receive?”
Home care/local services	“Dr will not home visit” – participant 12, baseline<br>“it would be a comfort if I could telephone a health worker for advice if needed.” – participant 179, baseline<br>“I do not have home visits.” – participant 229, 12 months<br>“Not enough doctors and specialists do home visits” – participant 254, 12 months<br>“Unable to see my GP on a regular basis” – participant 59, 18 months
Access to hospital services	“I need an operation and I cant get into the speacialist to be reviewed.” – participant 124, baseline<br>“Accessing hospital appointments doctors etc”– participant 164, 6 months<br>“The lack of hospital beds” – participant 126, 12 months<br>“Availability of specialists when it is convenient for me. Understaffing I suppose” – participant 403, 12 months
Individualis-ation of care	“where I am not included in my own care” – participant 28, baseline<br>“We have had no problems except for the meals during hospital stay. No real consideration for previous health issue of gastrectomy” – participant 228, 6 months<br>“No age specific advice regarding diet, exercise, ETC.” participant 122, 24 months
Coordination of care	“Not receiving documents to take to my GP from hospital.” – participant 52, baseline<br>“Because of the volume of patients communication between sections of care is poor and our personal information and paperwork are frequently been lost” – participant 141, baseline<br>“Because I see so many doctors information from a department to the others is not happening thus the doctors find it hard to treat me.” – participant 314, 12 months<br>“lack of communication between G.Ps hospital appts and allied health, results.” – participant 373, 12 months<br>“No communication between optometrist + G.P.” – participant 203, 18 months
Consistency of care	“Seeing GPs who are unfamiliar to me that are unable to make changes or assist” – participant 10, baseline<br>“Cannot find real reliable doctor. GPs push you in & out its like a cattleyard. Sometimes I go to clinic and you don't have same doctor.” – participant 97, baseline<br>“We have a different case manager each visit” – participant 196, 6 months<br>“not enough follow-ups” – participant 175, 12 months<br>“Everytime I received a different nurse who was unaware of previous discussions except in abare bones way” – participant 196, 12 months<br>“Those who come to dress my legs are not familiar with the wounds. They are not always the same nurse” – participant 68, 18 months<br>“the inconsistency of hospital care.” – participant 207, 18 months
Communic-ation	“Having multiple health professionals at bedside with no introductions & no communication from team to patient.” – participant 44, baseline

	"Lack of all information from doctors when admitted to hospital." – participant 46, baseline<br>"When doctor's don't explain things properly and thoroughly." – participant 204, baseline<br>"Not Knowing what's wrong with me" – participant 167, 6 months<br>"Not enough consultation time with some doctors whilst in hospital." – participant 394, 12 months
Waiting times	"Even when you have an appointment the time could still be another 3 hours or more." – participant 47, baseline<br>"Long waits at hospital & GP appointments. Why make an appointment if you need to wait" – participant 123, baseline<br>"Waiting is the biggest problem as in hospitals and seeing my GP usually takes 1-3 hours" – participant 133, baseline<br>"Waiting 4ever in the waiting room." – participant 151, 12 months<br>"Going to emergency and waiting for up to 12 hrs or so with uncomfortable chairs" – participant 260, 12 months<br>"waiting too long to see doctors in hospital." – participant 119, 18 months<br>"Waiting for hours in E.R when you feel very ill" – participant 141, 24 months
Healthcare provider qualities	"GP lack of understanding and lack of compassion" – participant 8, baseline<br>"Some attitude of the nurses" – particiant 32, baseline<br>"Traige nurses very rude." – participant 51, baseline<br>"sometimes I find doctors arrogant" – participant 155, baseline<br>"Current GP not very interested." – participant 274, 12 months<br>"G.P. not very Knowledgable" – participant 45, 18 months<br>"Some specialist lack of interest" – participant 212, 18 months
Transport/ parking	"Having to rely on people to transport me to appointment" – participant 138, baseline<br>"Getting to my local GP. Travelling is a problem as my back injury makes it difficult to sit for long periods." – participant 89, 6 months<br>"Limited parking spaces. As a pensioner we can't afford to pay parking at Sunshine." – participant 133, 6 months<br>"It is increasingly difficult to physically get to doctors appointments." – participant 254, 12 months<br>"Well I need transport to take me to appointments and so far I have to depend on taxis or a friend to take me" – participant 68, 24 months

Supplementary Materials Table 8: Comments made in response to question 3 – "What could be done to improve the healthcare you receive?"

Themes	Responses to question 3 "What could be done to improve the healthcare you receive?"
Home care/local services	"Dr could home visit" – participant 12, baseline<br>"Closer facilities for appointments" – participant 142, baseline<br>"Nurses to visit home." – participant 37, 6 months<br>"? Ability for health care services to come to the home" – participant 187, 6 months<br>"Nurse come when I ring healthlinks for advise" – participant 49, 18 months
Access to hospital services	"To have more doctors at hospitals" – participant 163, baseline<br>"I feel that some of the care I get is not enough. And trying to get referrals to specialist is very hard and help that I need." – participant 343, 12 months<br>"I think it would be nice to have more often access to the specialists" – participant 180, 18 months<br>"Build a hospital in the Western suburbs" – participant 126, 24 months
Individualis-ation of care	"Patients know their bodies and their feedback is very important in clinical decision making." – participant 45, baseline<br>"Better management of myself as a whole + treatment of many complex illness & conditions." – participant 126, baseline<br>"age specific exercise and diet programs (I'm not 80 years old)" – participant 122, 12 months<br>"More education x information age specific to my cardiac condition." – participant 122, 18 months
Coordination of care	"Better communication between services to make them easier to access." – participant 44, baseline<br>"Everyone is good except there is overlapping of services & no-one talks to each other. E.g-Xray done already but in hospital they do again." – participant 58, baseline<br>"Hospitals need to be able to access patient medical information quickly without needing to fish for it" – participant 213, 6 months<br>"One database that allows all health professionals to access your personal history. Many mistakes and inefficient time to repeat history to every health professional" – participant 230, 6 months

	"Better communication between medical and nursing staff." – participant 126, 12 months<br>"I would like my health care team (G.P. , Nurses, pharmacist) to communicate with each other about my treatment and care" – participant 190, 12 months<br>"To have a person to be able to contact that can then get the information needed. My GP does not appear to have the right contacts. I have on occasion needed alternative contact (private specialists)" – participant 180, 24 months
Consistency of care	"A service to contact when GP is away." – participant 104, baseline<br>"seeing same doctor at clinic" – participant 168, baseline<br>"consistency of healthcare" – participant 222, baseline<br>"Have one case manager per client" – participant 196, 6 months<br>"Ongoing care to make sure things stay on course is what I hope for" – participant 180, 12 months<br>"Keep to the same nurse and worker for each patient" – participant 196, 12 months<br>"outpatient have continuation with same specialist/dr, this is not currently with me/us." – participant 231, 12 months<br>"Increase consistency of hospital" – participant 207, 18 months<br>"Better follow up. In my particular situation I've had 3 hospital stays in the last ten years due to bone fractures. After the allocated physiotherapy, nobody has contacted me again." – participant 124 24 months
Communic-ation	"For doctors to talk to patients not around them." – participant 52, baseline<br>"Less bombarding with information is needed. Introduction at bedside very important." – participant 45, baseline<br>"Listening to what the patient has to say" – participant 189, 6 months<br>"To Know about what is our problems and to understand our health problems Example: I had heart treatment at the hospital but nobody told me what." – participant 199, 6 months<br>"Better communication between staff and patients at sunshine hospital" – participant 40, 12 months
Waiting times	"Need more staff and doctors and reduce waiting time" – participant 133, baseline<br>"Improve the waiting times in hospital. Recently I waited 2 hours on a ambulance stretcher with 3 ambos waiting with me, what a joke if I moved I could of topped over, they are so narrow." – participant 151, 6 months<br>"Cutting waiting lists and better service when a person attends emergency outpatients in public hospitals not waiting for very long hours to see a doctor." – participant 163, 12 months<br>"Keep the time of the appointments on time" – participant 119, 24 months
Healthcare provider qualities	"GP should take more time with patients." – participant 97, baseline<br>"Patients and understanding." – participant 45, 24 months
Transport/ parking	"More parking for disabled at hospitals" – participant 49, 6 months<br>"Maybe someone to drive to appointments when family members not available." – participant 37, 6 months<br>"Not pay parking fees" – participant 134, 12 months<br>"Establish more disabled parking spaces." – participant 283, 12 months<br>"I would appreciate to be transported to appointments" – participant 68, 24 months
